# Editorial: Data monitoring and triage practices during COVID-19: shaping the future of Public Mental Health

**DOI:** 10.3389/fpubh.2023.1174869

**Published:** 2023-04-11

**Authors:** Elke Van Hoof, Nele Van den Cruyce, Richard P. Bentall

**Affiliations:** ^1^Faculty of Psychological and Educational Sciences, Vrije Universiteit Brussel, Brussels, Belgium; ^2^Department of Psychology, The University of Sheffield, Sheffield, United Kingdom

**Keywords:** mental health, COVID-19, public health, fast response, innovation in public health

The COVID-19 pandemic could be considered the greatest psychological experiment ever conducted (1). Due to the lockdowns, extensive quarantines, and the chronicity of the imposed adjustments in everyone's life, COVID-19 has had a major impact on many aspects of our lives, including mental health. No one was prepared and no protocols existed to guide the management of the pandemic and the decisions of policymakers. This led to an explosion of data, approaches and processes, all with one goal: uncovering the necessary actionable insights as quickly as possible to keep the pandemic manageable and the consequences clear. The guest editors are therefore very grateful to the 35 contributors for sharing their insights. Although the crisis phase of the pandemic is over, the evaluation of the often innovative research techniques and review methods employed during this period can better prepare us for future sudden public health events.

Mental health is a broad concept ranging from distress to serious psychopathology with an adverse impact on the participatory capacity within society. Zhou et al. use a web of science core collection (WOSCC) to create order out of the chaos. Indeed, a multitude of scientific papers on the effects of the pandemic were published as it persisted, complicating the identification of actionable insights. A timely and effective intervention, however, depends on the ability to uncover actionable insights quickly. With the use of the WOSCC, it was possible to efficiently look at (1) where most publications take place, (2) which papers have had the most impact, and (3) which psychological problems are most common and how they were most studied. Anxiety and depression are especially common forms of psychological distress and so were studied extensively. Interestingly, nuances have been identified. For COVID-19 survivors, immune response measures have a direct link to depression and anxiety whereas panic about the uncertainty of the epidemic is a driver for mental distress in the general population. Young people who had to work outside their domicile presented with higher levels of anxiety and stress. In addition, inpatient mental healthcare was significantly reduced during the first period of the pandemic.

The imposed restrictions and quarantine methods were necessary to contain the infection but came at a high cost. As Gao et al. describe in their study of the impact of quarantine in the 3rd year of the pandemic in China, this cost also shows an evolution as the pandemic progressed and as people adapted despite continuing elevated levels of stress. While the rest of the world slowly learned to live with COVID-19, China opted to maintain strict quarantine rules. As a consequence, there was an immediate and prolonged effect on stress, depression, anxiety, poor sleep, unhealthy lifestyles and the development of mental disorders. The major predictor of the continually elevated stress response was financial insecurity, which impacted on emotions, physical responses, and behavior. Financial insecurity therefore seems inextricably linked to wellbeing. In addition, the authors also observed an important impact of age on emotional responses, with younger people most affected.

That young people were especially vulnerable to the impact of the pandemic was also demonstrated by Wang et al.. Their study investigated general psychological characteristics of college students in the phase of return to normality of the COVID-19 pandemic, and found elevated distress even after the return to normality. The mental health of the college student sample did not fully recover, with the main psychological and physical symptoms experienced including compulsive behavior, interpersonal sensitivity, anxiety, hostility, poor appetite, and insomnia.

From the results of the longitudinal study of Zielasek et al., carried out in North Rhine-Westphalia, Germany, it is clear that a reduction of case numbers of psychiatric inpatients—mostly in patients with affective and addiction disorders—was apparent. Even during the follow-up phases, the observed trend toward more acute cases, as indicated by diagnostic shifts toward more acute mental illnesses such as the psychotic disorders, and increased use of coercive methods were confirmed. Moreover, admission rates did not recover during the summer of 2020 nor in 2021. The strength of the Zielasek et al. study lies in the large and representative number of observed cases and the long observation period covering several phases of the pandemic. While psychiatric inpatient case numbers decreased, case severity and disease severity increased.

These findings are supported by the insights provided by the retrospective study of Di Lorenzo et al., carried out in Northern Italy. They emphasize that the most vulnerable and fragile people require more attention and care within emergency and community services. Both aggressiveness and socio-environmental maladjustment were observed more during the pandemic, suggesting a poorer adaptation response to the restrictions and uncertainty created by the pandemic. During the pandemic, people suffering from a severe psychiatric disorder represented the most vulnerable population.

Whereas the initial period of the pandemic generated a lot of research papers, the post-epidemic period did not. Countries and institutions need to further strengthen cooperation to identify actionable insights during the post-epidemic period. It is by combining both actionable insights from the outbreak and post-epidemic periods that timely and effective interventions can be identified for future (sudden) public health events.

## Author contributions

All authors listed have made a substantial, direct, and intellectual contribution to the work and approved it for publication.

## References

[1] Van Hoof E. Lockdown Is the World's Biggest Psychological Experiment - We Will Pay the Price. (2020). Available online at: https://www.weforum.org/agenda/2020/04/this-is-the-psychological-side-of-the-covid-19-pandemic-that-were-ignoring/ (accessed April 9, 2020).

